# Supplementary material for: Ferritin and Ferritin-to-Hemoglobin Ratio as Promising Prognostic Biomarkers of Severity in Acute Pancreatitis—A Cohort Study
Source: Biomedicines. 2024 Jan 4;12(1):106. doi: 10.3390/biomedicines12010106 (PMC10813292; doi:10.3390/biomedicines12010106)
Supplement: Supplementary file 1 [file biomedicines-12-00106-s001.zip › biomedicines-2784241-supplementary.pdf]

Supplementary Table S1 . The potential of SF, FHR and other biomarkers in predicting disease severity (MSAP), organ failure (OF) and mortality in the cohort

Parameter	MSAP			OF			Death		
	AUC (95%CI)	p value	Cut-off value(se; sp)	AUC (95%CI	p value	Cut-off value(se;sp)	AUC (95%CI	p value	Cut-off value(se;sp)
Serrum ferritin	0.640 (0.537;0.743)	0.011	202.81 (76%; 51%)	0.761 (0.652;0.870)	0.000	437.81 (71%; 75%)	0.802 (0.637; 0.966)	0.013	516 (83%; 74%)
FHR	0.666 (0.567;0.766)	0.002	13.94 (78%; 51%)	0.769 (0.659;0.879)	0.000	45.63 (61%; 88%)	0.794 (0.634;0.954)	0.016	51.58 (66%;86%)
Hematocrit	0.383 (0.281;0.485)	0.033	40.35 (40%; 39%)	0.426 (0.268;0.584)	0.292		0.462 (0.184;0.741)	0.755	
Leucocytes	0.598 (0.495;0.701)	0.074		0.651 (0.500;0.801)	0.031	14.79 (66%;74%)	0.627 (0.361;0.893)	0.295	
CRP	0.534 (0.428;0.640)	0.533		0.634 (0.504;0.765)	0.059	12.04 (95%;23%)	0.577 (0.390;0.765)	0.525	
Urea	0.594 (0.491;0.697)	0.087		0.798 (0.683;0.914)	0.000	42.50 (71%;75%)	0.787 (0.604;0.970)	0.018	42.50 (83%;70%)
Creatinine	0.627 (0.525;0.728)	0.021	0.98 (42%; 81%))	0.863 (0.760;0.967)	0.000	1.00 (85%;82%)	0.837 (0.663;1.000)	0.006	1.20 (83%;84%)
D-dimers	0.617 (0.505;0.730)	0.051		0.753 (0.626;0.881)	0.001	1507 (50%;90%)	0.842 (0.696;0.989)	0.021	
Fibrinogen	0.482 (0.372;0.592)	0.753		0.439 (0.291;0.587)	0.391		0.255 (0.070;0.440)	0.045	
Procalcitonin	0.623 (0.519;0.727)	0.025	0.16 (50%; 75%)	0.776 (0.658;0.895)	0.000	0.18 (85%;72%)	0.827 (0.714;0.939)	0.007	
LDH	0.551 (0.430;0.672)	0.413		0.796 (0.675;0.917)	0.000	408 (58%;91%)	0.731 (0.444;1.000)	0.084	

FHR-ferritin to hemoglobin ratio, CRP-C reactive protein; LDH-Lactate dehydrogenaze; AUC value-area under the curve; 95%CI-95% confidence interval, Se-sensitivity, Sp-specificity
